# Supplementary material for: Self-Referential Encoding on Modules of Anticodon Pairs—Roots of the Biological Flow System
Source: Life (Basel). 2017 Apr 6;7(2):16. doi: 10.3390/life7020016 (PMC5492138; doi:10.3390/life7020016)
Supplement: Supplementary file 1 [file life-07-00016-s001.pdf]

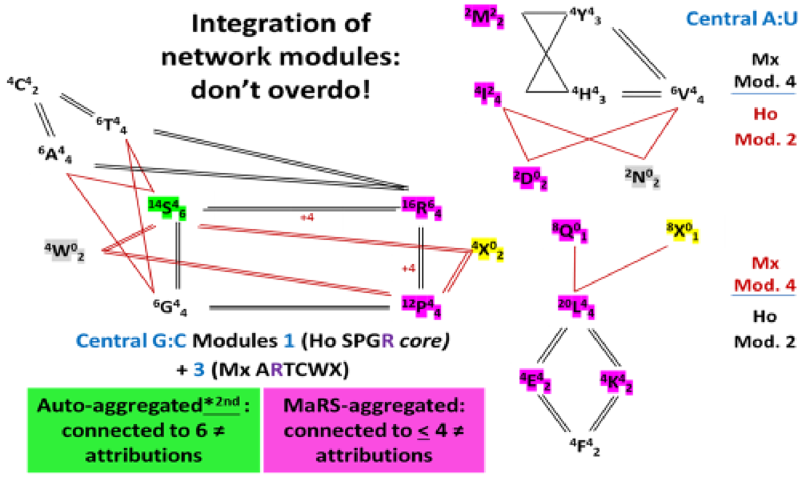

Supplementary information

1. Synthetase networks. (a) Networks formed by the tRNA pairs that correspond to an amino acid. Connections are in single lines, double or numbered; black for nonself-complementary, red for self-complementary triplets. Connections involving the anticodons corresponding to Stop (X) codons may be excluded. (b) Synthetases that are aggregated in the Multi-synthetase (MaRS) complex highlighted pink; the SerRS is self-aggregated, highlighted green.

It is suggested that SerRS self-aggregation is consequent to its too high number of connections to other amino acids, while the componentes of MaRS show lower number of connections. The number of aRS in MaRS is inversely proportional to the size of the subnetworks. Further details in [5,15].

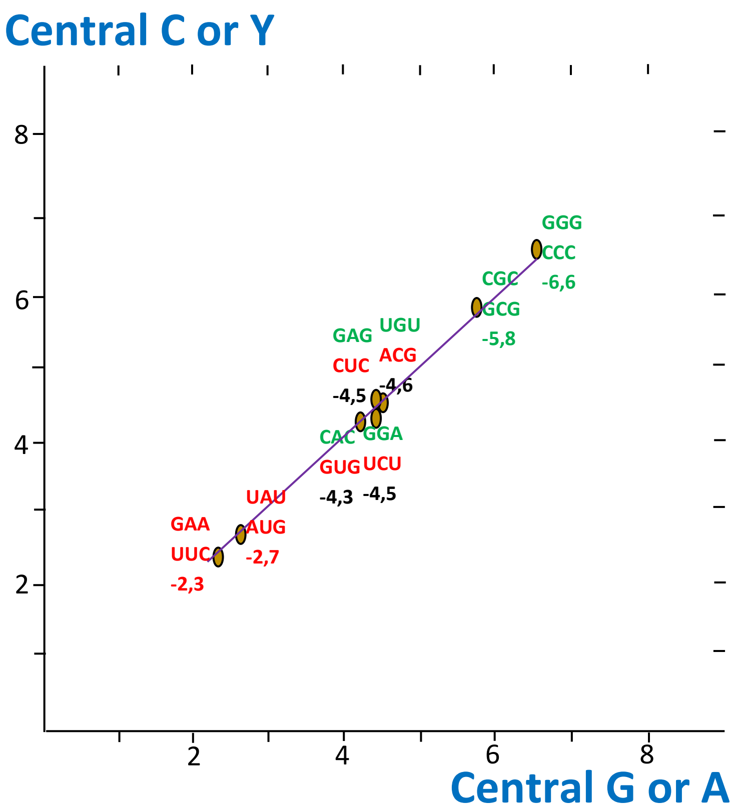

Supplementary information 2. Thermal stability of anticodon pairs. The highest value among all pairs that can be formed by the triplets in each box is shown. Triplets in green are for single-meaning boxes, in red for multiple-meanings boxes.
